# EEG-Triggered Functional Electrical Stimulation Therapy for Restoring Upper Limb Function in Chronic Stroke with Severe Hemiplegia

**DOI:** 10.1155/2016/9146213

**Published:** 2016-11-02

**Authors:** Cesar Marquez-Chin, Aaron Marquis, Milos R. Popovic

**Affiliations:** ^1^Rehabilitation Engineering Laboratory, The Lyndhurst Centre, Toronto Rehabilitation Institute-University Health Network, 520 Sutherland Drive, Toronto, ON, Canada M4G 3V9; ^2^Neural Engineering Laboratory, University Centre, Toronto Rehabilitation Institute-University Health Network, 550 University Avenue, Toronto, ON, Canada M5G 2A2; ^3^Rehabilitation Engineering Laboratory, Institute of Biomaterials and Biomedical Engineering, University of Toronto, Mining Building, 164 College Street, Room 407, Toronto, ON, Canada M5S 3E3

## Abstract

We report the therapeutic effects of integrating brain-computer interfacing technology and functional electrical stimulation therapy to restore upper limb reaching movements in a 64-year-old man with severe left hemiplegia following a hemorrhagic stroke he sustained six years prior to this study. He completed 40 90-minute sessions of functional electrical stimulation therapy using a custom-made neuroprosthesis that facilitated 5 different reaching movements. During each session, the participant attempted to reach with his paralyzed arm repeatedly. Stimulation for each of the movement phases (e.g., extending and retrieving the arm) was triggered when the power in the 18 Hz–28 Hz range (beta frequency range) of the participant's EEG activity, recorded with a single electrode, decreased below a predefined threshold. The function of the participant's arm showed a clinically significant improvement in the Fugl-Meyer Assessment Upper Extremity (FMA-UE) subscore (6 points) as well as moderate improvement in Functional Independence Measure Self-Care subscore (7 points). The changes in arm's function suggest that the combination of BCI technology and functional electrical stimulation therapy may restore voluntary motor function in individuals with chronic hemiplegia which results in severe upper limb deficit (FMA-UE ≤ 15), a population that does not benefit from current best-practice rehabilitation interventions.

## 1. Introduction

Stroke is one of the most common causes of disability [1, 2]. It can be the result of a rupture or infarction of the blood vessels supplying the brain, respectively, referred to as hemorrhagic or ischemic stroke. These events damage neighboring tissue and may lead to necrosis which can have important negative functional repercussions. The location and extent of the lesion often determine the nature and severity of the sequelae. Stroke often results in hemiplegia, in which one side of the body is paralyzed, when only one cerebral hemisphere is affected. This paralysis can have catastrophic effects on the independence and quality of life of individuals who have had a stroke. Impairments may range from very subtle (mild hemiplegia), in which individuals are able to continue performing movements to assist them in activities of daily living, to high (severe hemiplegia), in which the ability to move is greatly reduced or lost completely.

Despite the advances in the rehabilitation to restore voluntary movement after stroke, there are still individuals for whom effective intervention options are very limited. In particular, people with severe motor impairments may not be able to benefit from existing conventional therapies [1]. This is the case with stroke patients with severe upper limb deficit, measured by Fugl-Meyer Assessment Upper Extremity (FMA-UE) subscore of values equal to or lesser than 15. This situation is even more difficult for individuals with chronic conditions as recovery often takes place during the first months after having a stroke.

One of the few therapies available for individuals with severe hemiplegia following stroke is functional electrical stimulation therapy (FEST) [3]. Several reports suggest that this intervention is one of the most successful strategies to promote recovery after stroke and spinal cord injury [4–8]. Patients receiving FEST are asked to attempt a battery of specific movements and, after a few moments of unsuccessfully trying to perform the movement (e.g., 3 s–6 s), a therapist triggers a train of highly controlled electrical pulses to the paralyzed limb(s) producing the intended movement artificially. As patients recover voluntary function, the use of electrical stimulation is decreased gradually until it is completely discontinued.

The typical format for FEST is 40 one-hour sessions delivered three to five times per week for several months. In each session, patients engage in repetitive tasks focusing on their specific rehabilitation needs. For impaired upper limb movement after stroke, it is common to first focus on restoring reaching. Patients are asked to, for example, reach forward, sustain the arm extended for a few seconds, and then to retrieve their arm with all of the phases of movement assisted by electrical stimulation (i.e., reach, hold, and retract). Once recovery of reaching function is evident, grasping movements are practiced in subsequent therapeutic sessions, sometimes together with reaching movements and sometimes in isolation.

Brain-computer interfaces use brain signals to control electronic devices. Their operation does not require any voluntary movement, making this technology very promising to assist individuals with little or no ability to move. Populations that may benefit directly from this technology include individuals with amyotrophic lateral sclerosis, severe cases of cerebral palsy, and stroke resulting in total loss of motor function (e.g., brain stem stroke) [9]. Originally intended as an assistive device, this technology has been used to facilitate communication [10] and control computer cursors [11, 12], orthotic devices [13], and neuroprostheses [14–16], among other applications.

In the last decade, there has been an increased interest in the potential use of BCI technology to promote recovery of voluntary function after an insult to the nervous system, including stroke and spinal cord injury. One approach to use BCIs as part of a rehabilitation intervention to restore movement consists of training individuals to operate a BCI through motor imagery. Specifically, patients learn how to produce changes in the amplitude of the alpha (8 Hz–12 Hz) and/or beta (13 Hz–30 Hz) frequency ranges of their EEG by imagining voluntary movements [17–19]. These decreases in power, frequently referred to as event related desynchronization (ERD), can typically be observed during preparation, execution, and imagination of voluntary movement and have been used extensively for implementing BCI systems.

The main rationale behind training patients to produce ERD stems from observations in other forms of therapy, not including BCI technology, in which the EEG undergoes changes as functional improvement takes place; the brain activity can transform from a lack of response at the beginning of therapy to normalized ERD during movement at the time of discharge. The driving hypothesis of this intervention is that teaching patients how to produce normal motor-related EEG activity will result in improvements in voluntary motor function [20].

A recent report described an intervention consisting of motor-imagery-based BCI sessions held immediately before regular physical therapy sessions [21]. Participants in the intervention group were trained to produce voluntary power decreases in sensorimotor rhythms, recorded from their ipsilesional motor cortex, to control hand and arm orthosis. The participants in the control group did not use BCI system to control the hand and arm orthosis; instead it was activated randomly. Both the intervention and control groups received physiotherapy immediately after using the BCI. The “BCI therapy” produced a higher and significant increase in upper limb function at the end of the intervention compared to the control group.

More recently, a randomized control trial conducted by Pichiorri and colleagues [22] explored the use of BCI technology to monitor motor imagery during sessions conducted in addition to regular rehabilitation. In that study, 28 patients who had sustained a stroke a maximum of six weeks before the study underwent an intervention in which they were required to perform kinesthetic imagery of grasping and finger extension with their affected hands. The changes in power in the EEG activity resulting from the imagined movements by the participants in the intervention group (*n* = 14) controlled the movements of a life-size virtual hand projected on a screen placed over their hands. The individuals in the control group had a similar setup but excluded online control of the virtual hand. Their results showed greater functional outcomes for the intervention group, which also had an increased probability of achieving clinically significant changes.

A second method for incorporating a BCI into a rehabilitation intervention, and the one followed in this report, consists of activating an external device designed to facilitate movement of a paralyzed limb upon detecting the intention to move through analysis of brain activities during the rehabilitation sessions. The main hypothesis supporting this approach is that the paring of a motor command (produced when patients attempt to move) and relevant and correct sensory information (resulting from the artificially produced movement) will produce neuroplastic changes that in turn will result in improved voluntary motor function [20].

Control of orthotic and neuroprosthetic devices using BCI technology has been demonstrated several times [13–16], but exploration of the therapeutic effects of the combined technologies has only started recently with efforts focused primarily on the control of robotic rehabilitation systems. A recent randomized control trial conducted by Ang et al. tested the effects of a BCI-controlled robotic system to restore two-dimensional upper limb function [23]. Participants of that study attempted to reach to eight different targets with the assistance of the robotic device. The robot was triggered automatically or with mechanical cues for the control group and with a motor imagery-based BCI for the experimental group. Both groups experienced an improvement of arm's function after four weeks of treatment, with the intervention group requiring lower intensity therapy to experience the improvements (136 versus 1,040 repetitions for the intervention and control groups, resp.).

Daly and colleagues conducted an important study in which they used a BCI-controlled FEST for restoring voluntary finger's function [24]. The participant of that study had lost the ability to move her fingers individually as a result of a stroke she had sustained 10 months prior to the study. During the intervention, she was asked to attempt moving her fingers individually, and a BCI detected her intention to move, identified as a power reduction in the 13 Hz−30 Hz frequency range. The BCI, in turn, triggered a neuroprosthesis that produced the intended movement. The participant's ability to perform isolated finger movements increased after nine sessions.

We present here a proof of concept use of a BCI-triggered FEST for restoring upper limb's function. This combination of technologies was created as an enhancement to FEST in which activation of the stimulation was achieved by identifying changes in the EEG oscillatory activity indicating the attempt to move. The system was tested with a person with chronic hemiplegia (6 years after stoke) with severe upper limb deficit (FMA-UE ≤ 15), for whom all other forms of therapy had failed to produce any functional improvements in his ability to reach and for whom the expectations for recovery were low. This report describes our findings as well as some of the experiences that we had during this practical application of BCI technology.

## 2. Materials and Methods

### 2.1. Participant

The participant was a 64-year-old man who had sustained a right hemorrhagic stroke 72 months (six years) prior to his participation in this research study. Tomographic images confirmed an intraparenchymal hemorrhage deep in the right brain involving the subinsular and general capsule extending upward to the corona radiate. He had severe left hemiplegia with no residual movement. Prior to the stroke, he was left-handed. His arm and hand were at stage 1 on the Chedoke-McMaster Stages of Movement Recovery [25] and his FMA-UE subscore was 13. Prior to receiving BCI+FET, every other therapeutic intervention, including FET (without EEG activation) completed six months before this study, had failed to produce any clinically meaningful changes in his upper limb's function. He provided written informed consent to participate in this study, which was approved by the Toronto Rehabilitation Institute-University Health Network Research Ethics Board.

### 2.2. Experimental Setup

#### 2.2.1. Neuroprosthesis for Reaching

The neuroprosthesis for reaching was implemented with a four-channel programmable stimulator (Compex, Switzerland) [26], which was configured to produce the following reaching movements. 

*Forward Reaching and Retrieving.* It was produced by delivering stimulation to the* anterior deltoid* and the* triceps brachii* muscles (forward reaching) and posterior deltoid and biceps brachii (retrieve). 

*Reaching to the Mouth.* It was achieved by stimulating to the* anterior deltoid* and the* biceps brachii muscles* (forward reaching) and* posterior deltoid* and* triceps brachii* (retrieve).

*Lateral Reaching.* Lateral reaching included stimulating* biceps brachii* followed by* anterior* and* posterior deltoid* and finally by the* triceps brachii* (lateral reaching); retrieving was produced with stimulation of the* biceps brachii* muscle, interrupting stimulation to the deltoid muscle, and stimulating the* triceps brachii* again to produce extension of the arm.

In addition to these movements, the stimulation sequence for forward reaching and retrieving was used to produce* reaching to the right knee and retrieving*, and the stimulation synergy for reaching to the mouth was also used to facilitate* reaching to the right shoulder and retrieving*.

**Figure 1:**
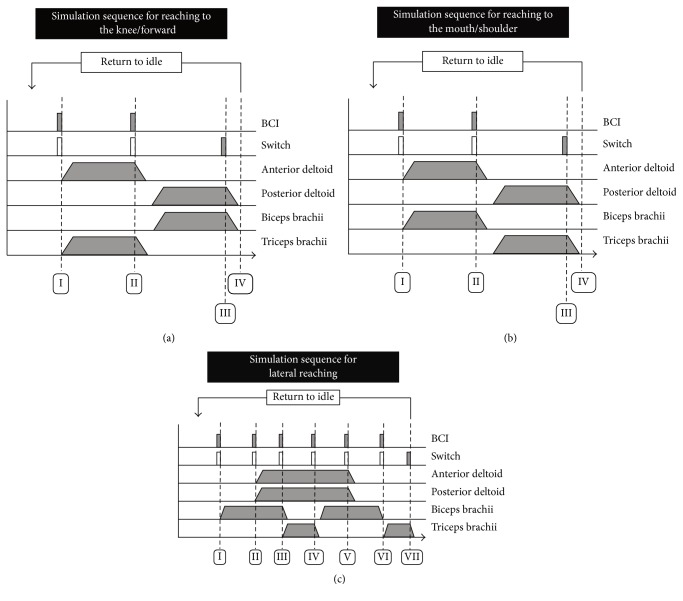
Stimulation sequences to facilitate (a) reaching to the right knee/forward, (b) reaching to the mouth/right shoulder, and (c) lateral reaching. For all sequences, activation of the BCI or switch would produce transition into the next phase of the movement. Empty rectangles in the “switch” component indicate optional activation.

The neuroprosthesis was designed to perform agonist and antagonist movements, each triggered in response to a command signal (e.g., external switch or BCI activation). For example, one BCI activation would facilitate a forward reaching motion of the arm and a second activation would trigger retrieval of the arm and its return to the starting (relaxed) position. Stimulation sequences for all of the facilitated movements can be found in Figure 1.

#### 2.2.2. Brain-Computer Interface

**Figure 2:**
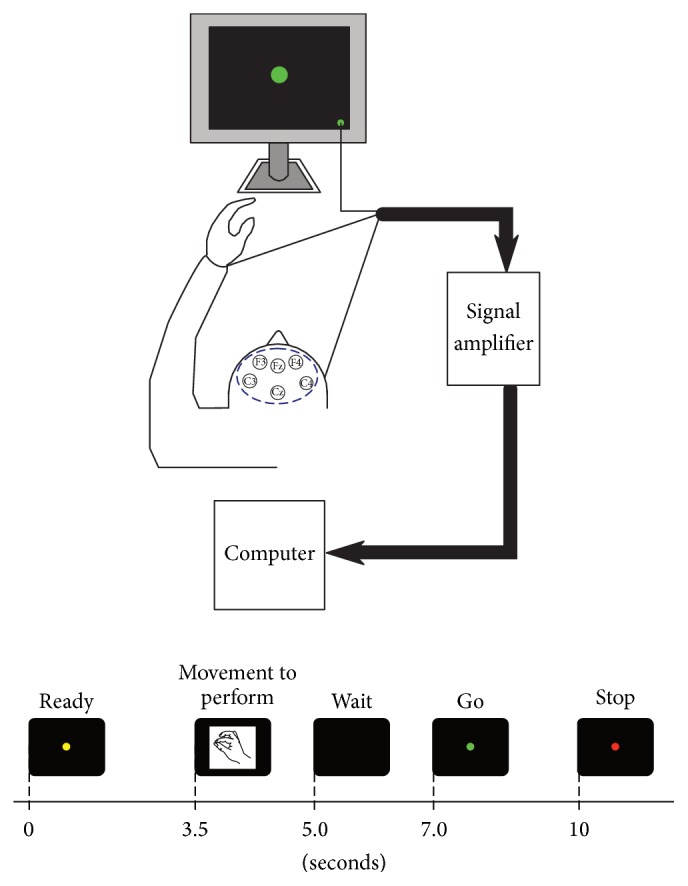
Experimental sequence used during calibration of the brain-computer interface. The participant sat comfortably in front of a computer monitor that provided cues indicating when to attempt specific movements with his paralyzed hand. These movements included precision pinch, hand closing (fist), lateral pinch, and hand opening and were presented at random.

The participant first completed a calibration session in which he was asked to attempt a series of six different hand movements with his left (affected) hand following a READY-GO-STOP cue (details provided in Figure 2). The movements included precision pinch, lateral and palmar grasps, and hand opening. Hand movements were chosen due to their common use in the configuration of motor-imagery-based BCIs. The numbers of repetitions for each movement are displayed in Table 1. At the same time, we recorded EEG from six different locations (F3, Fz, F4, C3, Cz, and C4 of the 10–20-electrode placement system) over premotor and motor cortical areas using the linked ears as a reference and the right mastoid as ground. No additional preprocessing was performed, as we wanted to create a BCI using a single electrode. The signals were digitized at 1,000 samples per second and band-limited between 0.05 Hz and 40 Hz. The EEG and the experimental cues were recorded using a SynAmps RT EEG amplifier (Compumedics, USA).

We segmented the EEG data into each one of the repetitions, which were aligned with respect to the GO cue. We then inspected each one of the repetitions for all movements visually and discarded any that was affected by interference, in which an incorrect movement was performed or in which the movement was not performed within the allotted time. All of the remaining repetitions for each movement were pooled together for further processing. After this, a spectrogram was generated for each one of the segments. To perform a quick inspection, we averaged all of the spectrograms from all of the electrodes, which revealed the potential locations and frequency bands displaying ERD. This preliminary step helped us in the process of generating ERD maps.

We generated ERD maps following the procedure described in [27]. Briefly, we applied a bank of band pass filters from 4 Hz to 30 Hz with overlapping bandwidths; the filters' center frequencies were separated by 1 Hz and had a bandwidth of 2 Hz. We squared every sample of every trial and applied a moving average filter (1 second) to smooth the resulting power signals, which we then averaged. The two seconds prior to the Go signal were averaged over time to obtain an estimate of baseline power for every sample and every spectral component. The remaining samples of the average power signal were expressed as relative changes (percentage) of the baseline power. We used *t*-statistic (*t* = 0.05) bootstrapping (500 bootstraps) to perform statistical validation of the observed changes in power. The process revealed Fz to be the site with the strongest ERD within the beta frequency band (18 Hz–28 Hz). This electrode and frequency band were used for the implementation of the BCI.

Once the suitable electrode placement and frequency range were determined, we created the BCI using a single EEG channel (Fz) recorded using a desktop biopotential amplifier (QP511, Grass-Telefunken, Germany) and a data acquisition system (USB-6363, National Instruments, USA) at a rate of 200 Hz prior to its acquisition. The EEG activity was band-limited between 10 Hz and 100 Hz and amplification gain of 20,000.

The BCI was implemented as a “brain-switch” that produced a monostable binary (on/off) control signal. This design supported the immediate integration of the BCI into FES therapy in which a switch is typically used to activate the electrical stimulation. The efficacy of “manually triggered” FEST has been demonstrated by several studies [4–8] and it is a current standard of practice within our clinical services. To do this, the root mean square (RMS) value of the EEG activity in the beta frequency range (18 Hz–30 Hz) was estimated every 125 ms. This value was then used to calculate a moving average that included the RMS values over the previous 500 ms (i.e., four estimates). This moving average was then processed with a simple line equation (i.e., *y* = *mx* + *b*) and the resulting modified moving average was displayed continuously on a computer screen in both graphical and numerical forms. This information was available to both experimenters constantly and it allowed for selecting the activation parameters for the BCI (all of them available through a graphical user interface). The same information also made it possible to monitor the BCI performance. The parameters *m* and *b* of the equation were adjusted heuristically by the second experimenter throughout each experimental session and allowed him to constrain the range of the signal (moving average). This range could be chosen arbitrarily at the preference of the experimenter.

**Figure 3:**
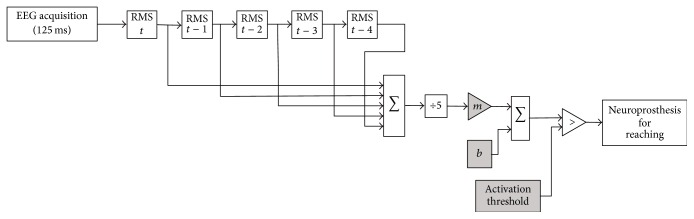
Implementation of the BCI. The BCI was implemented as “brain-switch” by comparing a moving average of the RMS values calculated over 625 ms decreased below an activation threshold. Elements in grey (*m*, *b*, and the activation threshold) were set heuristically by one of the experimenters throughout the experimental sessions using an online display of the RMS moving average as reference. The external switch is not shown in the figure.

The brain-switch was activated whenever the power in the resulting signal was sustained below an activation threshold for a prespecified duration. As with the parameters for the line equation used to constrain the range of the moving average, the activation threshold and latency values were also set manually by the second experimenter using the online display of the corrected RMS moving average. Adjustment of these two parameters changed the responsiveness of the BCI.  Figure 3 describes the implementation of the BCI used in this work, which was developed originally for creating BCI systems in environments with severe temporal and equipment restrictions (e.g., a limited number of electrodes), common in work conducted with electrocorticographic recordings [28]. Once the switch was activated, it was not possible to trigger it until the second experimenter “armed” it again. This made it possible for the participant to perform the motor tasks without a temporal restriction.

It is important to mention that while continuous BCI control of rehabilitation technologies provides a unique opportunity to monitor online the cerebral activity as related to motor attempt or imagery, a triggering approach does not imply that patients only attempt a motor task prior to the activation of the rehabilitation device (whether this is accomplished with a manual switch, electromyographic signal, mechanical cue, or BCI) but they rather continue with this attempt throughout the duration of each movement. In the context of FES therapy, the continued attempt to perform a voluntary movement is accomplished by asking patients to perform complete functional tasks (e.g., reach, grasp, retrieve, and release an object). In addition, there are several important factors that make FES unsuitable for online control. First, the movements produced by FES do not have the precision of other rehabilitation devices (such as a mechanized orthosis or a rehabilitation robotic system), which requires the intervention of an external agent (e.g., a therapist) to guide the limb in motion. Second, the dynamical behaviour of FES as it acts on the neuromuscular system has not been characterized successfully, severely restricting close-loop control FES applications.

#### 2.2.3. Integrated BCI and FEST System

**Figure 4:**
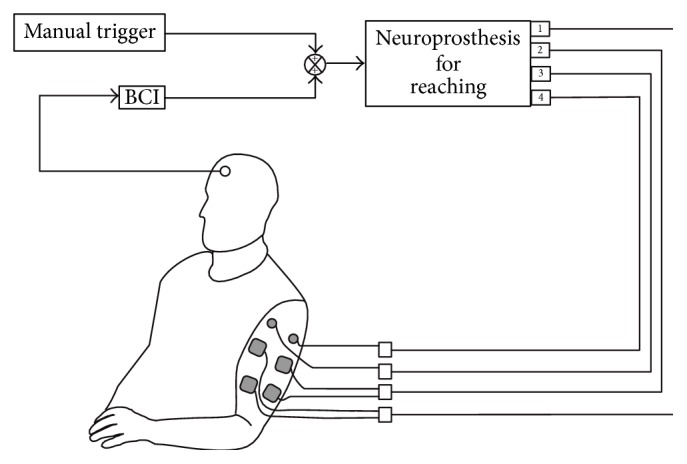
Illustration of the integrated BCI and FEST system. The electrical stimulation was delivered with a four-channel neuroprosthesis for reaching which could be triggered with a BCI or a manual switch. The BCI used the signal from a single electrode (positioned at location Fz of the 10–20 EEG electrode placement system), and the manual switch allowed for activating the stimulator whenever the BCI failed to identify the intention to move (note that ground channels for the anterior and posterior deltoid stimulation channels are not shown).

Integration of the BCI and FEST system was achieved with a single pulse (Transistor-Transistor-Logic (TTL) levels) that could produce a change in the state of the electrical stimulation sequence (Figure 1). In addition to the BCI, the stimulation sequence could also be triggered/controlled using an external switch. This was done to allow for bypassing the BCI in cases in which it failed to detect the intention to move. Operation with the manual switch was identical to that normally used in standard FEST (without integration with BCI).  Figure 4 displays the BCI+FEST system.

#### 2.2.4. Intervention

Two researchers delivered the EEG-triggered FEST. The first experimenter guided the movement of the arm, facilitated by the neuroprosthesis, while the participant actively attempted the movement. The researcher could also trigger the stimulation using a switch, bypassing the BCI system altogether. This was done to ensure that the stimulation was delivered when required (i.e., when the participant was attempting the movement) even if the BCI failed to identify the participant's intention to move. Manual activation of the stimulation is used commonly in FEST.

The second experimenter was responsible for making any necessary adjustments to the BCI throughout the duration of each session. These included increasing or decreasing the BCI activation thresholds and enabling or disabling EEG control of the neuroprosthesis for reaching. In addition, he also demonstrated the movements to perform.

The intervention consisted of 40 sessions, each lasting 90 minutes, delivered three times a week. The first 30 minutes of each session were used to prepare all the instrumentation required including placement of the EEG recording electrodes as well as donning and verifying the neuroprosthesis for reaching. The remaining 60 minutes were used to deliver the EEG-triggered FEST.

During the first week of the intervention, the participant received an explanation prior to the beginning of each trained movement including the trajectory to be followed, the starting and final positions of the hand, and the cue indicating the moment in which the movement was to be attempted. He was given the opportunity to practice several times the entire sequence of events until he indicated he was ready to start. It soon became evident that the participant had great difficulty remembering the sequence of actions to follow, which interfered with the intervention. For this reason, starting on the second week, one of the experimenters demonstrated the movement by performing it together with the patient at every repetition during the treatment.

#### 2.2.5. Sequence of Events

In each session, the participant attempted multiple repetitions of reaching to the mouth and right (opposite) shoulder and reaching forward and to the right (opposite) knee, as well as lateral reaching. All the movements started with the participant's left arm on his side and him sitting with good posture with both feet firmly planted on the ground. One of the experimenters cued the participant as to which specific movement to perform as well as when to attempt the movement. This was done by physically demonstrating the movement to perform, which the participant followed simultaneously. The movements were performed in phases with a brief pause between them. For example, in the case of reaching to the mouth, the participant would first attempt to touch his mouth with this left (affected) hand, he would then hold the hand in contact with his mouth for 2-3 seconds, and finally he would actively lower his arm back to the starting position. Once the arm was in the starting position, he was allowed to relax for a few moments (in which no electrical stimulation was applied), after which another cycle would start. Each movement was repeated between 20 and 30 times, and the participant was allowed to rest after completing all of the repetitions for each movement.

### 2.3. Outcomes Measures

We performed five assessments during the baseline, midpoint, and end of the intervention. These included Toronto Rehabilitation Institute-Hand Function Test (TRI-HFT) [29], Action Research Arm Test (ARAT) [30], Functional Independence Measure (FIM) [31], Self-Care Component of the Functional Independence Measure [31], and Fugl-Meyer Assessment Upper Extremity (FMA-UE) subscore [32]. The measured values are shown in Table 4. The Toronto Rehabilitation Institute-Hand Function Test was designed specifically to measure the changes produced during upper limb's rehabilitation using FEST, while the ARAT is an assessment of activity limitations of the upper limb. The FIM and FMA are two of the most widely accepted scales to measure changes during stroke rehabilitation.

## 3. Results and Discussion

### 3.1. BCI Configuration

**Figure 5:**
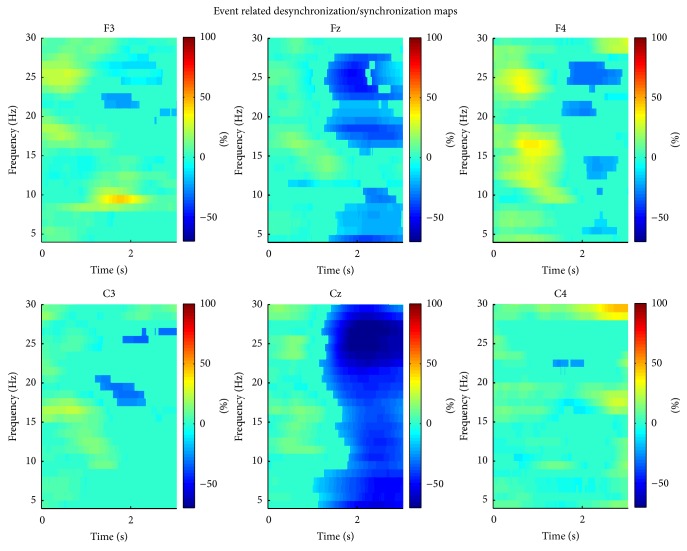
Event related desynchronization maps. We decided to use channel Fz for implementation of the BCI as it displayed behaviour resembling typical ERD (i.e., decreases in the alpha and beta ranges), compared to channel Cz, which showed a decrease of power across the entire spectrum. The plots display significant changes in power as a percentage of a two-second baseline period immediately before the Go cue (*t* = 0). Short increases in power were associated with ocular artifacts.

The most reactive electrode was found to be Fz, with decreases in power of 49.4% between the rest and the attempt to move (spectral differences and ERD maps are shown in Figure 5).

### 3.2. BCI Performance

Transition within each movement phase could be achieved by the BCI so movements with two phases (reaching to the mouth, shoulder, knee, and forward) required two instances of BCI activation, while lateral reaching required six instances of activation. A sample of 6 sessions was chosen to estimate the performance of the BCI. In total, 573 reaching tasks were included in the analysis of which 524 consisted of movements with two phases (i.e., reach and retrieve) and 49 were lateral reaching movements consisting of six different phases. Of the 1048 instances of BCI activation required for two-phase movements, 79% (414/524) and 63.9% (335/524) were successful for reaching and retrieving phases, respectively. Inspection of individual motor tasks revealed 81.3% as the highest BCI performance figure, which was achieved for movements targeting the mouth and shoulder during the reaching phase. In comparison, the lowest performance was recorded during the retrieve phase while reaching to the mouth with a value of 58%.  Table 2 provides details of the movements used to generate these results.

With respect to lateral reaching, 63.9% (188/294) of movement phase transitions were achieved with the BCI. Closer inspection showed that the highest accuracy was recorded during the first phase of the movement (performing elbow flexion starting with the arm relaxed on the side of the body) with a value of 93.9% (46/49). The poorest performance was recorded while extending the elbow with the arm abducted (phase III) with a success rate of 36.7% (18/49).  Table 3 shows BCI performance figures during lateral reaching.

The BCI responded well to the patient's attempted movements. This was evident by its activation almost exclusively during active periods in which he was instructed to reach with his arm (i.e., not during rest periods).

### 3.3. Changes in Arm Function

The Fugl-Meyer Assessment Upper Extremity subscore had a value of 13 points at baseline and of 19 at the end of the intervention. The FIM assessment had a baseline value of 104 points and 118 when the study was completed with the FIM Self-Care subscores registering 28 at baseline and 35 at the time of discharge. The Toronto Rehabilitation Institute-Hand Function Test Object Manipulation subscore and the Action Research Arm Test had a baseline value of zero and displayed no change at the end of the intervention.

### 3.4. Unique Aspects of the Integration of BCI and FEST Technologies

In addition to exploring the efficacy of a BCI-controlled FEST for restoration of upper limb's movements, this work also allowed us to identify elements that may be important for future integration of BCI and FEST technologies. There were unique technical challenges evident throughout the process of using BCI and FEST simultaneously. In addition, operation of a BCI by an individual with chronic severe hemiplegia resulting from stroke, with considerable cortical damage, presented a new set of challenges not commonly observed when BCI systems are tested on able-bodied individuals.

#### 3.4.1. Interference due to Voluntary Movement

One of the most obvious elements for the integration of BCI and FEST, as described here, was that the person using the BCI was actively attempting to move. This is different from one of the fundamental motivations of BCI development in which the technology was envisioned as a method to compensate for a severely limited or nonexisting ability to move voluntarily. We observed frequently muscle contractions unrelated to the required reaching motion, when the person was attempting/struggling to perform a movement. These movements often included pronounced facial gestures and bilateral shoulder contractions and were likely a manifestation of the physical and mental effort that the person was making while trying to move his affected arm. Although we did not measure EMG as part of our intervention, it was evident that the BCI failed to recognize the intention to move in these cases. The severity of this problem was reduced by asking the participant to keep his face and shoulders relaxed.

#### 3.4.2. Interference due to Electrical Stimulation

The electrical stimulation used by the neuroprosthesis to produce the movement may also produce electrical interference affecting the quality of the EEG recordings and subsequent operation of the BCI. The operation of our system did not appear to be affected by the electrical stimulation likely due to the fact that the stimulation pulses were delivered at 40 Hz, while the EEG frequency band used by the BCI was restricted to the beta activity (18 Hz–28 Hz). However, it should be noted that it is not uncommon to use asymmetric pulses, sometimes with discontinuities, which may still affect the spectral content of the EEG activity.

#### 3.4.3. Unobtrusive BCI

Another important feature of the presented work was that the operation of the BCI took a secondary role behind the delivery of FEST. In other words, we considered the delivery of FEST, and not the operation of the BCI, as the most important aspect of the intervention. One potential consequence that should not be overlooked is the level of motivation that the participant had, even at the end of the intervention, which is often not the case for regular FEST (i.e., not integrated with a BCI). This, in combination with the ease by which he could generate (FES assisted) reaching movements, allowed us to complete a much larger number of repetitions per session (approximately 35) than those typically practiced during standard FEST (approximately 10 per movement).

### 3.5. Potential Impact for Patients

The results observed in this case report suggest that triggering functional electrical stimulation therapy with the intention to move, using EEG indicators signaling motor intent, may produce restoration of reaching movements even 6 years (72 months) after having a stroke. It is also important to mention that the therapeutic effects may apply to individuals with severe hemiplegia (such as the case presented here), a population for which the options for therapy are very limited.

### 3.6. Weaknesses of the Study

The results show that, with the exception of three intermediate movement phases during lateral reaching, the BCI produced most of the transitions in the state of the electrical stimulation. We were pleasantly surprised by this finding along with the observed significant change in FMA-UE scores as well as the small increment in FIM Self-Care subscores. However, it is important to acknowledge the external manual switch as a confounding factor. The switch was included in our design as we were trying to create an enhanced version of FEST and we wanted to avoid a situation in which the operation of the BCI interfered with the actual FEST intervention. Future investigations of BCI-triggered FEST targeting multiple movements excluding the use of an external switch are warranted.

With respect to the manual selection of range-limiting and activation parameters, all values could be adjusted automatically in future versions of the system presented here. This could be accomplished using an online recursive calibration approach allowing us to obtain a measure of power at rest (baseline) and during attempting to perform a movement. However, it is important to point out that in the work described here activation of the BCI triggered a transition between different phases of the movement (e.g., forward reach and retract) which may require different processing including, for example, movement-phase-specific activation thresholds.

Another important potential weakness of the work presented is the demonstration performed by one of the experimenters to the participant. This, while it allowed the intervention to proceed without interruptions by the patient to clarify the type of movement to perform and the moment to execute it (both constant problems during the initial sessions), may have diverted the patient's attention towards the experimenter and away from the execution of the motor task. However, it should be mentioned that a fundamental therapeutic component of FEST is the use of functional tasks during the therapeutic intervention, which requires that patients be fully engaged in the motor task. In addition, verbal, visual, and tactile cues are commonly used in rehabilitation to facilitate the initiation of movement.

Also important is to discuss the use of different movements for creating the BCI (i.e., grasping movements) and those facilitated by the FES (i.e., reaching movements). Hand movements were selected due to their common use for the development of ERD-based BCI system. Although suitable for this initial proof-of-concept work, the next versions of our work will try to ensure that configuration of the BCI is performed using the same movements targeted during rehabilitation.

## 4. Conclusions

A 64-year-old man with chronic severe left hemiplegia resulting from stroke received 40 90-minute sessions of BCI+FEST (brain-computer interface triggered functional electrical stimulation therapy) to restore reaching function (forward, mouth, knee, opposite shoulder, and lateral). Every other intervention that the patient received since having the stroke had failed to produce improvements in his upper limb's function. The BCI used a single EEG channel and triggered individual phases of each of the reaching tasks, when it detected the individual's intention to move.

## Figures and Tables

**Table 1:** Movements performed for BCI configuration.

Movement	Number of repetitions
Precision pinch	20
Hand closing (fist)	26
Lateral pinch	34
Hand opening	24

Total	104

**Table 2:** Performance for movements with two phases (reach and retrieve).

	Movement target				Total
	Mouth	Shoulder	Forward	Knee	
Number of repetitions	150	123	158	93	524

	Performance figures during reaching				
Expected BCI activation	150	123	158	93	524
Recorded BCI activation	122	100	118	74	414
Successful BCI activation	81.3%	81.3%	74.7%	79.6%	79.0%

	Performance figures during retrieving				
Expected BCI activation	150	123	158	93	524
Recorded BCI activation	87	72	118	58	335
Successful BCI activation	58.0%	58.5%	74.7%	62.4%	63.9%

**Table 3:** Performance for lateral reaching.

Number of repetitions	49	
Total expected BCI activation	294	

	Phase I	Arm relaxed to elbow flexion

Expected BCI activation	49	
Recorded BCI activation	46	
Successful BCI activation	93.9%	

	Phase II	Shoulder abduction with elbow flexion

Expected BCI activation	49	
Recorded BCI activation	40	
Successful BCI activation	81.6%	

	Phase III	Elbow extension with shoulder abduction

Expected BCI activation	49	
Recorded BCI activation	18	
Successful BCI activation	36.7%	

	Phase IV	Shoulder abduction with elbow flexion

Expected BCI activation	49	
Recorded BCI activation	22	
Successful BCI activation	44.9%	

	Phase V	Shoulder adduction with elbow flexion

Expected BCI activation	49	
Recorded BCI activation	24	
Successful BCI activation	49.0%	

	Phase VI	Elbow flexion to arm relaxed

Expected BCI activation	49	
Recorded BCI activation	38	
Successful BCI activation	77.6%	

**Table 4:** Performed assessments.

	Baseline	Midpoint	Discharge
TRI Hand Function Test Object Manipulation subscore	0	0	0
Action Research Arm Test	0	0	0
FIM Self-Care subscore	28	35	35
FIM total	104	118	118
Fugl-Meyer Assessment Upper Extremity subscore	13	18	19
